# Visual rehabilitation: visual scanning, multisensory stimulation and vision restoration trainings

**DOI:** 10.3389/fnbeh.2015.00192

**Published:** 2015-07-27

**Authors:** Neil M. Dundon, Caterina Bertini, Elisabetta Làdavas, Bernhard A. Sabel, Carolin Gall

**Affiliations:** ^1^Department of Psychology, University of BolognaBologna, Italy; ^2^Centre for Studies and Research in Cognitive Neuroscience, University of BolognaCesena, Italy; ^3^Medical Faculty, Institute of Medical Psychology, Otto-von-Guericke University of MagdeburgMagdeburg, Germany

**Keywords:** hemianopia, vision restoration training, audio-visual training, visual scanning training, neural plasticity

## Abstract

Neuropsychological training methods of visual rehabilitation for homonymous vision loss caused by postchiasmatic damage fall into two fundamental paradigms: “compensation” and “restoration”. Existing methods can be classified into three groups: Visual Scanning Training (VST), Audio-Visual Scanning Training (AViST) and Vision Restoration Training (VRT). VST and AViST aim at compensating vision loss by training eye scanning movements, whereas VRT aims at improving lost vision by activating residual visual functions by training light detection and discrimination of visual stimuli. This review discusses the rationale underlying these paradigms and summarizes the available evidence with respect to treatment efficacy. The issues raised in our review should help guide clinical care and stimulate new ideas for future research uncovering the underlying neural correlates of the different treatment paradigms. We propose that both local “within-system” interactions (i.e., relying on plasticity within peri-lesional spared tissue) and changes in more global “between-system” networks (i.e., recruiting alternative visual pathways) contribute to both vision restoration and compensatory rehabilitation, which ultimately have implications for the rehabilitation of cognitive functions.

## Introduction

Homonymous visual field defects (HVFD) are among the most serious deficits after cerebral artery stroke and traumatic brain injury (TBI) in adults (Bouwmeester et al., 2007). HVFD result from damage to the visual pathway behind the chiasma, i.e., posterior brain regions including the optic tract, optic radiation and visual cortex, typically with either complete or partial loss of visual perception in one half of the visual field. HVFD affect both eyes in a homonymous manner, i.e., the loss of vision is on the same side of the visual field in both eyes. HVFD affect 20–30% of individuals who suffer cerebrovascular infarction (Rossi et al., 1990).

In approximately 70% of cases, patients with HVFD present with parafoveal visual field sparing of the central five degrees which enables them to fixate centrally (Kerkhoff, 2000). Nonetheless, HVFD patients suffer enduring difficulties in their everyday lives, such as impaired reading, navigation, visual exploration (Bouwmeester et al., 2007), visual cognition and motor-control (Kerkhoff, 2000). Vision loss is also a key issue in more general neuropsychological diagnosis and rehabilitation of cognitive functions; for example, both psychometric testing and computer-based training methods require sufficient vision to detect and identify stimuli (such as words or numbers, e.g., Tsai and Wang, 2015). HVFD can therefore compromise occupational rehabilitation, further disabling work or domestic life (Kerkhoff, 2000; Bouwmeester et al., 2007; Gall et al., 2009). In fact, vision loss may also lead to wrong diagnoses, such as neglect or agnosia (Serino et al., 2014).

In adult patients with HVFD, a certain amount of spontaneous recovery of the visual field may occur in the first 2–3 months post-lesion (Zhang et al., 2006). However, such spontaneous recovery is usually partial and only occurs in 20–30% of cases (Zihl and von Cramon, 1985). After this early recovery phase, further spontaneous improvements are rare (though exceptions have been reported, see Poggel et al., 2001). Spontaneous HVFD recovery tends to occur primarily in the visual periphery (Kerkhoff, 2000), which may be explained by either the cortical magnification factor (CMF; i.e., the peripheral visual field is processed by a lower number of neurons, but with larger receptive fields, compared to the foveal space; Çelebisoy et al., 2011; Harvey and Dumoulin, 2011; Wu et al., 2012) or by recruitment of the undamaged retino-collicular extra-striate pathway, which preferentially processes stimuli from the periphery of the visual field, in addition to processing the perception of movement (for review, see Sabel et al., 2011b).

In general, however, there is no additional spontaneous recovery beyond the first few months, and the HVFD is considered to be permanent. As a consequence, since the late 70–80 s the combined efforts of neuropsychological research and clinical practice have sought to achieve HVDF improvements in the post-acute stage of recovery through visual rehabilitation (for early rehabilitation studies, see Ben-Yishay and Diller, 1981, 1993; Ducarne and Barbeau, 1981; Ducarne et al., 1981; for a review, see Coubard et al., 2014). In this perspective, the term visual rehabilitation (Kerkhoff, 2000; Zihl, 2010) refers to all the rehabilitation strategies aiming to improve hemianopic patients’ independent living and quality of life, promoting functional restitution of the impaired visual function (restoration approaches), the acquisition of compensatory strategies relying on the intact functions (compensatory approaches) or the adaptation of the environment to the patient’s impairment, through artificial devices (substitution approaches).

The substitution approach does not rely on the plastic cortical reorganization properties of the lesioned brain, but aims at replacing the lost vision by artificial (usually optical) means. Among others, substitution methodologies include prosthesis (Hossain et al., 2005), optical prisms that project the unseen visual sector into parts of the intact visual field (Bowers et al., 2008, 2012; O’Neill et al., 2011), and reading aids connected to television or personal computers (Virgili et al., 2013).

On the other hand, compensation of the visual field loss might be accomplished by improving the gaze field by training patients to make saccadic eye movements toward the blind hemifield (Zihl, 1995, 1999; Nelles et al., 2001; Roth et al., 2009). Indeed, the fundamental goal of compensatory approaches is to enhance saccadic responses and other oculomotor parameters. In contrast, restoration methods aim at increasing the sensitivity of residual tissue and expanding the visual field itself, by activating residual structures of the damaged visual field to strengthen their neuronal activity and synaptic plasticity. The latter can be accomplished by vision training of areas of residual vision (ARV) or by applying non-invasive brain current stimulation (reviewed by Sabel et al., 2011b). Findings from these studies challenge the prevailing view that post-acute vision loss is both permanent and unchangeable.

Compensatory and restorative approaches have become popular in the last two decades thanks to the continued expansion of findings demonstrating experience-dependent plastic reorganization in the human visual system (Karmarkar and Dan, 2006; Martins Rosa et al., 2013). Since the pioneering studies in animal models (Wiesel and Hubel, 1965; Gilbert and Wiesel, 1992; Eysel et al., 1999), recent data on humans have revealed several forms of plasticity, such as perceptual learning (Gilbert et al., 2001; Fahle and Poggio, 2002) and long-term adaptation (Webster, 2011), providing evidence that plastic reorganization remains largely functional in the adult human visual system. Notably, early studies revealed that tactile image projections could effectively substitute vision for object recognition, demonstrating cortical plastic reorganization in the visual system of blind individuals, since early studies revealing that tactile image projections could effectively substitute vision for object recognition (Bach-y-Rita et al., 1969). Furthermore, a growing amount of evidence demonstrates improved visual performance and enhanced activation of visual areas using non-invasive human-machine interfaces (i.e., sensory substitution devices), which transform visual information into auditory or tactile representations (Abboud et al., 2014; for a review: Maidenbaum et al., 2014).

In the present paper, we discuss three main contemporary paradigms of vision rehabilitation: Visual Scanning Training (VST), Audio-Visual Scanning Training (AViST), and Vision Restoration Training (VRT). We will present the rationale which has shaped these paradigms and detail their respective methodologies. We will then report the outcomes of each treatment, drawing upon the body of available literature and criticisms offered by alternative viewpoints. Finally, we will discuss emergent neuroimaging and electroencephalographic data which are beginning to uncover the neural mechanisms underlying these treatment approaches.

## How Compensation and Restoration Approaches Differ

The main difference between compensation and restoration is that the former primarily aims to recruit alternative unaffected brain regions that can play a compensatory role in the visual process, whereas the latter relies on the notion that stimulation of areas of partial injury (represented by partially functioning regions of the visual field) might induce synaptic plasticity and thus improvement to lost visual functions. Restoration techniques aim at modifying the visual system itself by lowering the threshold of perception. There has been a vigorous and controversial debate about whether vision restoration is possible at all (Sabel and Trauzettel-Klosinksi, 2005). On the one hand several authors have presented evidence that vision restoration is possible by behavioral training that activates areas with lowered perceptual thresholds or inconsistent light detection (Kasten et al., 1998, 2006; Hyvärinen et al., 2002; Sabel et al., 2004; Sabel and Trauzettel-Klosinksi, 2005; Sahraie et al., 2006; Bouwmeester et al., 2007; Vanni et al., 2010). On the other hand, the fundamental concept of a functional restoration of vision has been vigorously opposed by different authors who argue that compensatory methods are the only way to help HVFD patients (Reinhard et al., 2005; Glisson, 2006; Pelak et al., 2007; Roth et al., 2009). In fact, also approaches such as flicker training (belonging to the field of vision restoration) were reported as ineffective, though the authors failed to apply the stimulation adequately since residual vision was not tested at those positions that were used for flicker training (Roth et al., 2009). During flicker training, stimuli are typically presented deep within the blind field. However, the experimenter failed to ensure the presence of residual vision within the blind field before defining the positions for flicker stimulation. In other studies, flicker training consistently results in increased detection sensitivity even deep within the blind field (Hyvärinen et al., 2002; Sahraie et al., 2006; Vanni et al., 2010). However, as our discussion below shows, compensation and restoration are not mutually exclusive concepts and both are worthy of further study.

## Compensation Training by VST

The term VST is used in this review to cover different types of unisensory (visual) eye movement training. The term “unisensory” refers to tasks which draw upon a single sensory modality, i.e., vision, in order to enhance visual functions; there is no ancillary recruitment of other senses (such as auditory or somatosensory cues as discussed below).

**Figure 1:**
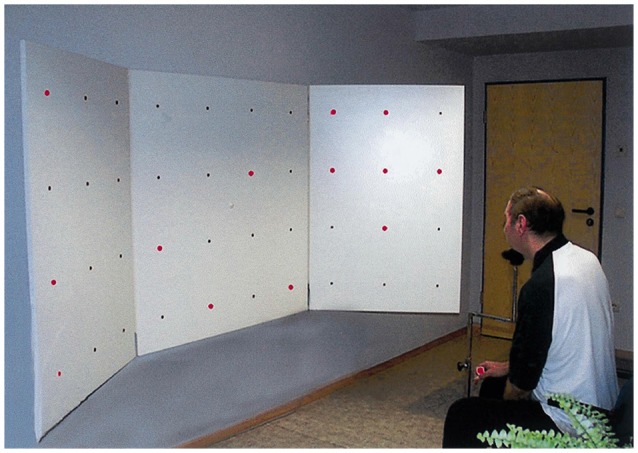
**Visual search task in Nelles et al. (2001).** Patients were presented with simple red lights that were equally distributed across the board in four horizontal lines with ten lights in each line. The task was to identify a target stimulus (square of four lights) by exploratory eye movements with restricted head movements (with permission from Elsevier).

Compensatory approaches are oculomotor strategies which aim to train saccadic eye movements; thus, they do not specifically target the size of the scotoma but rather the field of view (i.e., the part of the visual scene that can be scanned by eye movements). Typically, this is accomplished by training patients to voluntarily (and consciously) explore arrays of visual stimuli—usually on computer screens (Zihl, 1995, 1999)—but stimuli may also be presented in far vision (Nelles et al., 2001; Figure 1). The rationale is to bias patients towards their blind hemifield in order to compensate for the restricted field of view resulting from the scotoma (Zihl, 1995). Indeed, HVFD patients do not spontaneously compensate for visual field loss, usually showing defective oculomotor behavior. Typically, patients perform more fixation and refixations compared to healthy controls and they show saccades with decreased amplitudes towards the hemianopic side, resulting in longer time of visual exploration (Meienberg et al., 1981; Pambakian et al., 2000). Therefore, by moving the eyes back and forth more often, the intact visual field sector then catches a greater area of the visual scene, increasing the so-called “field of view” (not to be confused with visual field enlargements achieved by vision restoration techniques). VST trains patients to make adaptive saccades into the affected blind field and systematically scan the visual scene in order to compensate for their loss by making better use of the intact visual field (Gassel and Williams, 1963; Ishiai et al., 1987). For example, if the vision loss is on the right, patients are taught to move their eyes more frequently to the right so that they may see objects more easily with their intact, left visual field sector.

Total training duration for VST is typically around 1 month, consisting of daily 1 h sessions. After about 5–6 weeks of VST, patients generally report improvements in scanning accuracy, exploration times and daily life activities (Kerkhoff et al., 1992, 1994; Zihl, 1995, 2000; Nelles et al., 2001; Pambakian et al., 2004; Verlohr and Dannheim, 2007; Mannan et al., 2010). However, notwithstanding these improvements, some concerns have been raised regarding the treatment’s net-effects. When the size of the HVFD remains constant, which is the case in most VST studies, scanning more often towards the hemianopic side, e.g., to the right, results in a shift of the intact temporal visual field sector moving temporarily out of the field of view. In other words, developing a positive bias of moving the eyes more often towards the right means an automatic negative bias of not seeing objects on the left. Furthermore, increasing the volume of eye scanning also increases the integration load of the brain, as a larger amount of moving retinal images needs to be fused into a coherent object or motion; this increased load may be a problem for brains that suffer temporal processing and integration deficits (Schadow et al., 2009; Poggel et al., 2011).

Comparing the results of different VST studies is challenging, as different authors have experimented with different training protocols, varying either in the degree of cognitive demands or in the eccentricity of the field of view within which targets are presented. For example, Zihl (1995) introduced a simple visual detection task, requiring adult patients (mean age: 44 years) to shift their eyes towards the hemianopic field, after an acoustic signal, in order to find the visual target, i.e., a spot of light. Head movements were restricted as typical in these early VST studies (Zihl, 1995, 1999). Thus, early VST protocols only train oculomotor behavior for visual search in the range of near vision and the training area was reduced to a computer or television screen. A second phase of the training required patients to perform visual search tasks in a large stimulus array (52° × 45°). At the end of both phases of the training, visual search time markedly decreased and visual scanning behavior was better organized, showing a number of saccades and fixations similar to the one exhibited by healthy controls. In contrast, no change in the visual field size was observed. In a study by Nelles et al. (2001), VST was performed on a large display (3 m wide training board at a distance of 1.5 m) and adult patients (mean age: 59 years) were asked to systematically scan the board horizontally, to train saccades with restricted head movements. Performances after training revealed improved detection rate and reaction time to visual stimuli presented at the training board, when exploratory eye movements were allowed. In contrast, when exploratory eye movements were not allowed, no detection improvement was found. As in Zihl’s (1995) study, VST resulted in a compensation of the HVFD without any measurable restoration of visual fields. It is worth noting that compensatory oculomotor strategies in HVFD patients also rely on working memory resources (Hardiess et al., 2010). Therefore, the training protocol recommended to patients should be chosen in accordance with the patient’s performance level and availability of cognitive resources. The current trend is to develop visual tasks and training protocols with varying processing demands, presented on realistic, large field stimulus displays with unrestricted head movements, with simultaneous measurements of both head and eye movements. Papageorgiou et al. (2012) recently observed effective compensatory gaze patterns in patients with HVFD performing a more complex real life task (dynamic collision avoidance); these patterns include increased exploratory eye and head movements towards the blind side.

In summary, VST techniques offer a relatively short intervention with positive outcomes. Studies using similar VST paradigms to those described above (Zihl, 1995; Nelles et al., 2001) have found similar improvements in visual scanning behavior and visual detection with exploratory eye movements (Kerkhoff et al., 1994; Pambakian et al., 2004). More importantly, VST can also reduce the self-reported perceived disability of adult HVFD patients in daily activities, such as bumping into obstacles and crossing the street, thereby showing a transfer of training effects to ecological measures (Kerkhoff et al., 1994; Nelles et al., 2001). These effects can also promote successful return to work (Kerkhoff et al., 1994). Interestingly, age does not appear to be a critical factor in predicting the outcome of VST training: indeed, both older and younger adult patients achieve the same rehabilitation outcomes with the same amount of training (Schuett and Zihl, 2013). In addition, the beneficial results of VST remain stable as far as 8 months post treatment (Kerkhoff et al., 1994; Nelles et al., 2001).

## AViST

AViST is the latest development in the field of compensatory interventions for visual field defects. In contrast to the classic compensatory interventions of VST which are unisensory, i.e., using only visual stimuli, AViST is multisensory. One advantage of AViST over VST, therefore, is the multisensory nature of the stimulation. Indeed, multisensory experience can adaptively maximize the sensory input options available to the organism when perceiving and localizing stimuli in the space.

Pioneering studies on animals (Stein and Meredith, 1993) have revealed the neurophysiological basis of multisensory integrative processes at the single neuron level, showing enhanced neural responses in the multisensory neurons of the superior colliculus (SC) when auditory and visual stimuli are in register, i.e., when presented in spatial and temporal coincidence (spatial and temporal principles of multisensory integration). Such enhanced neural responses are super-additive, i.e., the response to the combination of auditory and visual stimuli exceeds the sum of the responses to the single sensory stimulus (i.e., multisensory enhancement). Moreover, the effectiveness of the modality-specific signals is a major determinant of multisensory enhancement, with pairs of unisensory weakly effective stimuli resulting in more robust enhancement of the multisensory neuronal activity (i.e., the inverse efficacy principle; Stein and Stanford, 2008). A pivotal role in supporting the integrative processing in the SC has been demonstrated by heteromodal associative cortices in the cat (i.e., AES, rLS; Jiang et al., 2001; Jiang and Stein, 2003). In line with this finding, the inferior parietal (Dong et al., 1994) and intraparietal cortices (Colby et al., 1993; Duhamel et al., 1998; Schlack et al., 2002) have been suggested as sites of convergence of sensory information from many different modalities in primates. Additionally, imaging studies in humans have confirmed the involvement of the SC and posterior cortical areas, including the temporo-parietal and posterior parietal cortices, in mediating audio-visual multisensory integration (for a review: Calvert, 2001; Stein and Stanford, 2008).

Crucially, converging evidence also suggests the presence of multisensory benefits at the behavioral level, both in animals’ orienting responses (Gingras et al., 2009) and in a wide range of perceptual tasks in humans (for review see: Alais et al., 2010). In particular, behavioral studies on healthy participants have shown that multisensory integrative mechanisms can improve both detection (Frassinetti et al., 2002; Bolognini et al., 2005a; Bertini et al., 2008; Leo et al., 2008a; Maravita et al., 2008) and localization (Hairston et al., 2003; Lovelace et al., 2003; Alais and Burr, 2004; Bolognini et al., 2007; Leo et al., 2008b; Bertini et al., 2010) of audio-visual pairs consisting of degraded unisensory stimuli. Interestingly, repeated exposure to coincident audio-visual pairs of stimuli effectively facilitates visual learning (Kim et al., 2008) and enhances activation in extrastriate cortical areas (Shams and Kim, 2012). More importantly, audio-visual integration can increase perceptual performances in patients with unisensory defects, such as HVFD or neglect (Frassinetti et al., 2005), low vision (Targher et al., 2012) or auditory localization deficits (Bolognini et al., 2007). In particular, visual detection of stimuli presented in the blind field of patients with HVFD was significantly improved by the presentation of spatio-temporal aligned audio-visual stimuli, while no improvement was found when stimuli were presented in spatial disparity or temporal asynchrony (Frassinetti et al., 2005).

As a consequence, the AViST model posits that audio-visual multisensory integration can be a useful resource for rehabilitation of unisensory visual defects, and that the recruitment of the retino-colliculo-extrastriate pathway, which is usually spared after post-chiasmatic lesions causing HVFD, can compensate for the loss of visual perception. In line with the inverse effectiveness principle, impaired unisensory processing in HVFD might be improved by multisensory stimulation. Indeed, multisensory neural circuits, retaining their responsiveness to cross-modal stimuli, might constitute the neural basis for the compensation of impaired sensory modalities (Làdavas, 2008).

**Figure 2:**
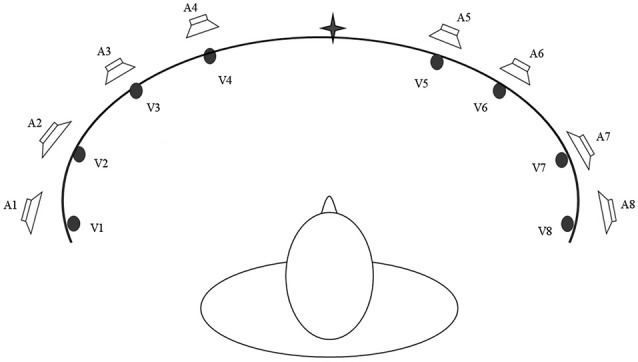
**A schematic bird’s eye view of the apparatus used for the Audio-Visual Scanning Training (AViST), depicting the location of visual (V1–V8) and auditory (A1–A8) stimuli.** Stimuli are positioned at 8, 24, 40 and 56 visual degrees into both the left and right visual field in an ellipse shaped apparatus (200 cm wide, 30 cm height).

Based on these multisensory principles, Bolognini et al. (2005b) developed a training protocol to determine whether systematic stimulation of the visual field over the course of a period of training with combined audio-visual stimuli would lead to long lasting amelioration of unisensory visual orientation and detection deficits in patients with chronic post-chiasmatic lesions. Training was administered by seating patients centrally at the concave face of an ellipse shaped apparatus in a horizontal arc in a dimly lit and sound-proof room (Figure 2). Eight piezoelectric loudspeakers were positioned along the eye-line at 8, 24, 40 and 56° of eccentricity towards the right and left side to present auditory stimuli (bursts of 100 ms of white noise). In addition, eight red LED lights were placed on the exact same position of the loudspeakers to present visual stimuli for 100 ms. During training, patients first fixated upon the central point of the arc (± 30° of vertical eccentricity respectively for inferior and superior quadrantopia), then explored the visual field by moving their eyes, not their heads. In each block, the patients were instructed to search for visual stimuli being presented alone (unisensory condition) or coupled with an auditory stimulus (multisensory condition). Lone auditory stimuli constituted a catch trial condition. Participants were encouraged to move their eyes left or right along the median line, exploring the visual field. Afterwards they were instructed that the sound might sometimes (but not always) be predictive of the location of the light. To boost oculomotor exploration of the hemianopic visual field, a greater proportion of stimuli was presented in the blind visual field—so that the patients learned to respond more easily to that side over time. Patients responded with button press after detection of a visual stimulus. Training duration was approximately 2 weeks at a rate of 4 h/working day. Patients completed training by achieving a hit-rate of >50%, i.e., consistently above chance level, in the unisensory visual condition in the blind field for one entire training session.

After the treatment, HVFD patients (average lesion age: 12 months; mean age: 57 years) showed an increase of visual detections (without fixation requirement), improvements of visual search and reading abilities and a reduction in self-perceived disability in daily activities (such as bumping into obstacles, crossing the street, finding objects in an ecological environment). The improvements were stable at a 1 month follow-up (Bolognini et al., 2005b). In addition, Passamonti et al. (2009) revealed that the same treatment was also effective in a different sample of patients (average lesion age: 58.16 months; mean age: 43 years) in improving oculomotor parameters during visual search and reading tasks. In particular, all patients reported an improvement in oculomotor exploration after treatment, which was characterized by fewer fixations and refixations, faster and larger saccades, and a reduced scanpath length, leading to a shorter exploration time, compared to pre-treatment performances. Similarly, training significantly affected oculomotor reading parameters, reducing both progressive and regressive saccades. Further, the treatment drove improvements with respect to the specific reading impairments observed in both left and right hemisphere—damaged patients (Leff et al., 2000); saccadic amplitude increased for right hemianopic patients and the number of saccades during the return sweep reduced in left hemianopic patients (Passamonti et al., 2009). Notably, as in the Bolognini et al. (2005b) study, the training promoted a reduction in self-perceived disability in daily life activities, confirming a transfer of the effects of the training to ecological environments. In this study, improvements were stable at follow-up assessment, 1 year after training.

However, it is worth noting that the improvements were only seen in tasks where patients were able to use eye movements to compensate for the loss of their vision. Indeed, no amelioration was found in the visual detection task where patients fixated the central fixation cross. The discrepancy between the results of the tasks where exploratory eye movements were allowed and where they were not allowed (when central fixation was required) suggests that the improvement in visual perception induced by the training is not due to an enlargement of the visual field, but rather to an activation of the visual responsiveness of the oculomotor system, reinforcing orientation towards the blind hemifield.

Crucially, the amelioration cannot be attributed to a mere habituation effect whereby the training simply encourages saccades towards the hemianopic field; indeed, a similar training protocol, using unisensory visual stimuli instead of multisensory audio-visual stimuli, yielded no improvements in a control patient group (Passamonti et al., 2009). This finding suggests the multisensory nature of the stimulation is the critical factor inducing amelioration. It could also be argued that having two stimuli rather than one increases the overall attentional salience, i.e., the magnitude of training stimuli reaching the senses, in the hemianopic field and thus pulls the attention spotlight in this direction. In other words, the important component of the training is not its multisensory nature, but rather it simply works by having an increased magnitude of stimulation in the hemianopic field via two stimuli rather than only one. Though this cannot yet be ruled out conclusively, the observation that perception does not improve unless stimuli are spatially and temporally aligned as described above, i.e., conforming to multisensory principles, suggests that aggregation of attentional salience may not be a sufficient explanation of treatment effects. Interestingly, significant visual exploration and improvements in oculomotor parameters also occur in patients with recently acquired occipital lesions, after a similar compensatory audio-visual training in the acute post-stroke phase within 24 weeks after brain injury (Keller and Lefin-Rank, 2010). In line with previous evidence (Passamonti et al., 2009), patients showed greater oculomotor (i.e., an increased number and amplitude of saccades towards the hemianopic field) and compensatory visual scanning improvements after audio-visual stimulation, compared to unisensory visual stimulation, providing further evidence of the clinical advantages of a multisensory exploration training.

We propose that the training was effective at integrating sensory inputs from different sensory modalities related to the same external event, consequently enhancing the efficiency of eye saccades to the presentation of the visual stimulus. More specifically, it appears that repetitive audio-visual stimulation of the hemianopic field mediates an exogenous shift of multisensory attention in this direction, which strengthens oculomotor mechanisms to scan the hemianopic field more efficiently. Since patients with visual defects tend to direct the focus of their attention to the intact hemifield (Sabel et al., 2011b), the auditory cue interacting with visual input reverses this tendency by inducing an exogenous shift of multisensory spatial attention towards the blind hemifield.

We further propose that recruitment of the spared retino-colliculo-extrastriate pathway might drive this effect. Converging evidence reveals the pivotal role of the SC in integrating audio-visual spatio-temporal coincident stimuli in humans (Calvert et al., 2001; Bertini et al., 2008; Leo et al., 2008a; Maravita et al., 2008), and the relevance of temporo-parietal and posterior parietal cortices in mediating covert and overt orienting behavior towards audio-visual stimuli (Meienbrock et al., 2007; Bertini et al., 2010; Nardo et al., 2014). In addition, recent findings have provided evidence that after disruption of the primary visual cortex, the retino-colliculo-extrastriate pathway is functionally and anatomically spared and could foster orienting responses toward visual stimuli presented in the blind field (Tamietto et al., 2012). Intensive multisensory stimulation during training could have enhanced the activity of this network and allowed the implementation of more efficient oculomotor patterns due to stronger links between SC and other higher order cognitive areas, such as the frontal eye fields, which contribute to oculomotor planning (Arikuni et al., 1980; Barbas and Mesulam, 1985). In line, connections between the SC and the frontal eye fields are thought to join a neural circuit mediating spatial attention shifts (for a review: Krauzlis et al., 2013).

In summary, initial evidence suggests that combining different sensory modalities represents an effective training for visual field defects. However, further studies are needed to explore the neural underpinnings of the compensation of visual field defects after AViST.

## Vision Restoration by VRT

According to the residual vision activation theory (Sabel et al., 2011b), cerebral visual injury is usually not complete, and some structures are typically spared in or near the area of damage. Such areas of spared neurons lie in different places in the brain: (i) in penumbral areas of partial damage at the border of the lesion; (ii) in islands of surviving tissue dispersed within the lesion; (iii) extrastriate pathways unaffected by the damage; and (iv) down-stream, higher-level neuronal networks. The functional status of these structures is likely to be compromised because the damaged visual system suffers an enduring tripartite handicap: (i) partially damaged areas have fewer neurons; (ii) they lack sufficient attentional resources; and (iii) neurons in areas of partial damage have poor firing synchrony. Residual structures therefore no longer contribute (or contribute rather little) to every-day vision and their silencing through non-use further impairs their synaptic strength.

The VRT approach posits that such partially spared regions of cortex are functionally represented by ARV. More specifically, visual field deficits do not produce an absolute, binary split between areas of total blindness and areas of intact vision (the black-and-white view of vision loss), separated by a sharp and clearly definable visual field border. Rather, visual field defects actually comprise: (i) areas of total blindness; (ii) areas of consistent (normal) visual detection; and (iii) areas where visual detection performance is present but inconsistent.

Typically, visual field charts are based on applying standard static near-threshold perimetry using a low resolution stimulus presentation, presented in a monocular fashion. However, due to the rather low resolution, these perimetric tasks are not sensitive enough to decipher smaller regions of inconsistent (partial) visual detection. Standard near-threshold testing methods predominantly delineate areas of vision loss vs. intact vision while the topography of the ARV which is typically located at the border zone along the defect remains unclear. By increasing the number of test positions it is theoretically possible to gain the missing information by means of near-threshold perimetry. However, this procedure is not used in clinical settings since it results in a tremendous increase in test duration. Supra-threshold high resolution perimetry (HRP) has therefore been used to test detection of light stimuli binocularly within a dense grid of stimulus presentations. This greater sensitivity has assisted with both the characterization of distinct regions of ARV, and the evaluation of treatment effects (Kasten et al., 1998).

The presence of ARV and VRT effects have been considered to be artifacts of inaccurate diagnostic measurements resulting from poor fixation because of excessive eye movements (Reinhard et al., 2005; Glisson, 2006). However, good fixation abilities are a prerequisite for VRT. Furthermore, ARVs can be very well replicated across repeated measurements and eye tracker recordings show that the standard deviation of the mean fixation point in patients with homonymous hemianopia is about 0.82° horizontally and 1.16° before VRT (Kasten et al., 2006).

Increasing evidence gathered with HRP suggests that patients with cerebral visual injuries have ARV of varying sizes, typically at the transition between areas of total blindness and areas of normal visual detection, i.e., at the visual field border, or in islands of residual vision within the areas of total blindness. To plan VRT sessions, these ARV regions are first identified so that they can then be activated to enhance the function of underlying partially spared neuronal tissue. It is hypothesized that VRT re-engages these residual structures by repetitive stimulation and activation of ARV. The residual vision activation theory posits that when a certain minimum number of neurons remains connected to their target structure, they can lay the foundation for neuroplastic reorganization via synaptic plasticity and subsequent functional improvement following VRT in HVFD patients with chronic lesions (Sabel et al., 2011b) just as in normal perceptual learning (Li et al., 2004; Fahle, 2005). Animal studies are compatible with this hypothesis; a relatively small number of intact cells (10–20%) can support spontaneous recovery up to 70–80% normal performance in simple visual detection in rats within 2–3 weeks post-lesion (Sautter and Sabel, 1993).

VRT can induce visual perception improvement at any time after the lesion, at all ages and in all types of visual field impairments after retinal or brain damage (such as stroke, brain trauma etc.). Concerning the influence of age on VRT outcomes, Kasten et al. (2000) could not reproduce earlier results from a VRT pilot study pointing to an effect of age on visual field enlargement (Kasten and Sabel, 1995). In fact, in a large clinical observational study with a sample size of more than 300 subjects, patients aged 65 years and older benefited more from VRT than younger patients (Mueller et al., 2007).

If and to what extent vision restoration can be achieved is a function of the amount of residual tissue and its activation state, and of the status of global neuronal networks (Sabel et al., 2011b). Sustained improvements require repetitive stimulation which, depending on the method, may take days (non-invasive brain stimulation; Fedorov et al., 2011; Sabel et al., 2011a; Gall et al., 2013; Schmidt et al., 2013) or months (behavioral training with VRT; Kasten et al., 1998; Poggel et al., 2004; Sabel et al., 2004). By becoming re-engaged in every day vision, (re)activation of ARV by VRT outlasts the training period, thus contributing to lasting vision restoration and improvements in quality of life (Gall et al., 2008).

**Figure 3:**
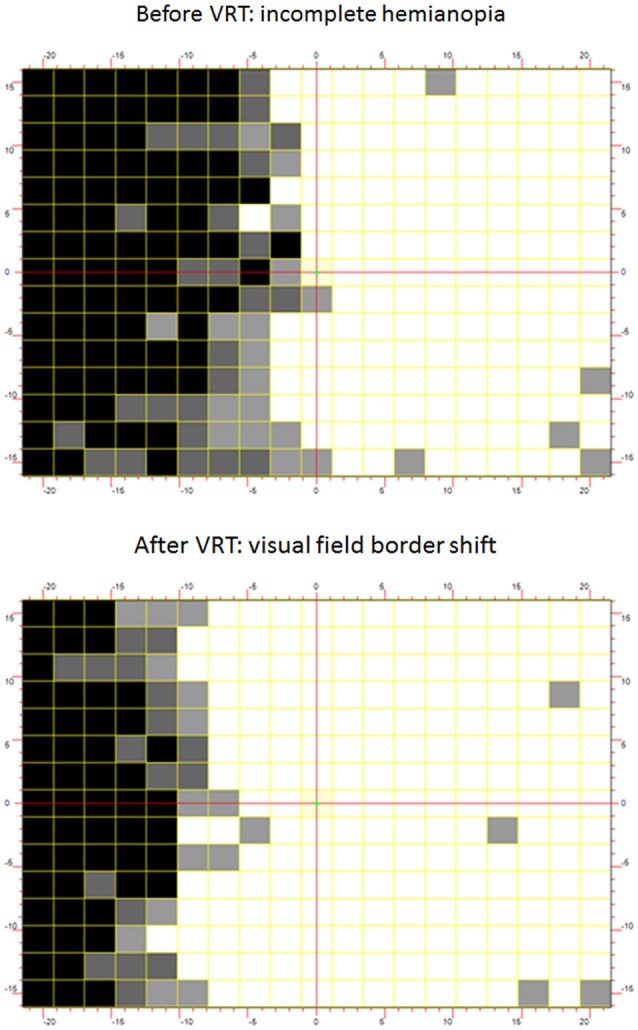
**(Upper panel) Baseline-Vision Restoration Training (VRT) visual field chart.** To assess the visual fields with high-resolution computer-based perimetry, suprathreshold stimuli are presented at random from which simple detection charts can be created. By superimposing results of repeated tests, intact visual field sectors are shown in white and black represents regions of absolute blindness where no stimulus detections occurred. Gray areas reveal areas of residual vision (ARV) where response accuracy is inconsistent. Area of residual vision correspond to relative defects in standard-automated perimetry and may be interpreted as representing partial damage where only some cells remain connected with their target structure. Thus, partial structure leads to partial function. **(Lower panel)** The chart depicts the Post-VRT result in the same subject. The visual field defect has resolved mainly within the area of residual vision at baseline.

Drawing on this new understanding, methodologies were developed to accurately identify ARV, and train these areas according to individualized protocols of VRT. Kasten et al. (1997) described a collection of computer programs utilized in VRT. Participants’ visual field defects are first evaluated using HRP performed on a computer screen in a dimly lit room. During HRP, small light spots appear with luminance well above physiological thresholds in random order in a dense grid of 25 × 19 stimulus positions surrounding a central fixation point. Subjects respond to any perception of these light spots. Fixation is ascertained by the accurate response to an isoluminant change in color of the fixation point. The tested visual field covers up to 20 visual degrees both vertically and horizontally. Figure 3 shows a HRP visual field chart in which white squares graphically illustrate normal visual detection performance. In contrast, black squares reflect areas of zero detection and gray areas indicate ARV where responses were present, but inconsistent (Figure 3).

After delineating residual function (i.e., identifying the ARV), the training procedure is individually adapted to the patient’s specific deficit pattern. VRT is developed to specifically target ARV with the goal of strengthening these structures by repetitive activation. This is achieved by presentation of localized “static” stimuli along the visual field border or “dynamic” stimuli which appear first in either the blind or intact field and move to the nearest point in the ARV (Kasten et al., 1997). Patients respond to each perceived stimulus via button press. The training protocol is adaptive—when the detection performance exceeds a pre-determined point, typically, greater than 90% correct responses, the computer programme advances to the next level by presenting stimuli deeper within the blind field. Training usually takes place in the participant’s home for 30–60 min per day, for a period of at least 6 months. The central outcome measure after VRT is light detection accuracy change as observed in HRP (Figure 3) or standard-automated perimetry procedures. In sum, the treatment is effective at driving improvements with the majority of patients, by an average visual field border shift of 5° of visual angle (Kasten et al., 1998). Improvements have also been reported to generalize to some extent to visual exploration tasks (Kasten et al., 1998; Reinhard et al., 2005). Notably, patients also report subjective improvements in ecological environments (Kasten et al., 1998; Julkunen et al., 2003; Reinhard et al., 2005). The positive outcomes of VRT appear stable up to 23 months after the training (Kasten et al., 2001; Julkunen et al., 2003; Marshall et al., 2008; Poggel et al., 2010). According to different reviews (Bouwmeester et al., 2007; Sabel et al., 2011b), some patients do not benefit from VRT (33%), moderate improvements are seen in about 33% and large field expansions in another 33% percent of the patients. Of a total number of 37 publications, all but three confirmed the efficacy of the VRT paradigm (Sabel et al., 2011b). The non-confirmatory studies, however, suffered from methodological limitations; one limitation is that the training duration was too short and the training stimuli too small to achieve clinically relevant effects (Balliet et al., 1985). In another case, insensitive methods of measuring visual detections were employed (Sabel et al., 2004; Reinhard et al., 2005). The third study failed to focus the therapy on ARV but rather used a simple flickering stimulus which was presented deep in the blind field where no residual structures were present (Roth et al., 2009). Thus, the latter study actually did not use the typical VRT-treatment protocol as other studies did.

As mentioned above, some authors have raised doubts about the validity of VRT (e.g., Reinhard et al., 2005; Glisson, 2006; Bouwmeester et al., 2007; Pelak et al., 2007). The argument is that plasticity is not possible in the visual system and that visual field improvement after VRT is merely a compensation artifact in disguise, i.e., that increased eye movements explain the visual field improvement. However, this compensation artifact claim can be rejected for several reasons (Kasten et al., 2006; Sabel et al., 2011b). Firstly, it stands to reason that if training improvement was merely a function of random lateral fluctuating saccades, there could not be a systematic shift of the border in one direction, as consistently documented. On this point, some authors have suggested that the treatment effect is systematically biased by the lateral saccades, i.e., preferential scanning towards the hemianopic side in evaluation tests. However, this response bias would require patients to be able to predict the (randomly chosen) position of the transient evaluation stimuli during post-treatment testing which is logically and practically impossible. In addition, good fixation ability is a prerequisite for training, which dismisses propositions that results are confounded by eccentric fixation. Further, treatment does not appear to change the position of the blind-spot (for more detail, see Sabel et al., 2011b).

Recent evidence also adds to these logical arguments. For example, training does not always cause a shift of the entire visual field border. Quite often shifts only occur in one sector of the border (for example in the upper visual field). Further, post-training visual field border shifts for patients with concentric visual field loss, as in glaucoma, typically move in a ring-like fashion in all directions towards the periphery (Gudlin et al., 2008; Sabel and Gudlin, 2014). Additionally, eye movements are not directionally specific (before or after treatment), blind spot positions do not appear to shift, and eye movement amplitudes after VRT actually decrease, suggesting a post-training improvement in fixation quality (Kasten et al., 2006). More recent studies have also availed of eye-movement adjusted retinal charts observing new stimulus detections after VRT in previously blind areas of the visual field (Sabel et al., 2011b). Thus, while the eye is not expected to be exactly at fixation at all times—since microsaccades are a normal repertoire of visual perception (Ahissar and Arieli, 2012)—both experimental evidence and logical reasoning rules out eye movements as explaining improvements following vision restoration, though they are always a possible source of variability and, respectively, error, in visual field testing. In summary, many independent studies show the efficacy of VRT in achieving visual field improvements (Kasten et al., 1998; Sabel et al., 2004; Poggel et al., 2006; Henriksson et al., 2007; Gudlin et al., 2008; Marshall et al., 2008; Romano et al., 2008; Ho et al., 2009; Halko et al., 2011; Plow et al., 2012).

VRT does not appear to drive change solely in the visual cortex. Ho et al. (2009) used retinotopic mapping when analysing residual function after VRT and observed responses in extrastriate areas above the calcarine sulcus. Functional Magnectic Resonance Imaging (fMRI) studies of the Blood Oxygen Level Dependent (BOLD) change following VRT have also observed increased post-training activations in anterior cingulate and dorsolateral frontal cortex, in addition to the recruitment of higher order visual areas in the occipitotemporal and middle temporal regions (Marshall et al., 2008). Henriksson et al. (2007) further observed ipsilateral representation of the trained visual hemifield in different cortical areas, including the primary visual cortex. Thus, the emerging evidence suggests that VRT drives plastic cortical reorganization both at the within-systems and the network level, i.e., training drives activation increases not only in occipital regions but also in wider distributed attention networks. In fact, this concept of global network change has most recently been demonstrated by electroencephalography (EEG) network analyses in optic nerve patients treated with non-invasive brain current stimulation which also improved patients’ visual fields (Bola et al., 2014).

## Discussion

The evidence presented in this review supports the idea that visual rehabilitation, defined as the promotion of improvements in independent living and quality of life, can be achieved with adult HVFD patients using either VST, AViST or VRT (Kerkhoff, 2000; Pambakian et al., 2005; Schofield and Leff, 2009; Zihl, 2010; Trauzettel-Klosinski, 2011; de Haan et al., 2014; Goodwin, 2014). The reviewed studies suggest that VST and AViST induce long term improvements in patients’ visual exploration abilities, promoting a more organized pattern of fixations and refixations and increasing the amplitude of the saccades (Zihl, 1995; Nelles et al., 2001; Bolognini et al., 2005b; Passamonti et al., 2009). In contrast, VRT reportedly induces an average visual field border shift of 5 degrees of visual angle (Kasten et al., 1998). Although VST, AViST and VRT promote different visual functions, all three approaches have been demonstrated to generalize the positive outcomes observed with clinical measurements also to daily life activities (Kerkhoff et al., 1994; Kasten et al., 1998; Nelles et al., 2001; Julkunen et al., 2003; Bolognini et al., 2005b; Reinhard et al., 2005; Passamonti et al., 2009; Dundon et al., in press).

Since on first appraisal VST, AViST and VRT appear to seek different outcomes, they seem to fall under separate and specific rehabilitation models—restoration (VRT) vs. compensation (VST), or a compensation/restoration hybrid (AViST).

In general terms, restorative therapies aim at improving the magnitude of visual function, while VST and AViST compensate for the visual field loss. However, in terms of the neural mechanisms underlying the two approaches, each of these two ostensibly disparate treatment methodologies may well draw on both, local and distal, cortical reorganization mechanisms. This suggests that in case of visual rehabilitation, concepts of restoration and compensation can be both fluid and reciprocal. Broadly speaking, neuroplastic changes can be indexed into two categories, delineated by the associated lesion proximity and overall diffusivity of cortical reorganization. The first model, within systems plasticity, targets reconnection of damaged neural circuits proximal to, or within islands of spared tissue surrounded by the lesion (Robertson and Murre, 1999; Sabel et al., 2011b). The second model, network level plasticity, refers to recruitment of more widespread processes of cortical reorganization, such as homologous areas in the intact hemisphere or alternative spared pathways subserving the visual function (Sabel et al., 2011b), in order to compensate for loss of specific neuronal function (Bola et al., 2014).

Within the context of visual rehabilitation following brain injury, within-systems plasticity would target functional restoration of partially spared cortex within or near the striate or extra-striate lesion, i.e., repairing neurons to re-engage them in their previous function; this model would appear to best describe the plastic reorganization driven by VRT. Conversely, network level plasticity recruits alternative networks to compensate for the lost function within a different specific area, i.e., the visual cortex; this model would appear to best describe the change elicited by VST and AViST. Apparently, the above axis appears to neatly compartmentalize the three treatments presented in this paper. However, the emerging findings from studies attempting to document the underlying substrate change driven by these therapies seem to contradict such a simple binary hypothesis.

Concerning VRT, fMRI studies have revealed effects within wider distributed networks, i.e., BOLD changes occur not only in the visual cortex, but also in extrastriate areas (Henriksson et al., 2007; Marshall et al., 2008; Ho et al., 2009). In a similar vein, the notion that treatment improvements driven by VST are exclusively driven by compensatory eye movements has been challenged by some recent experimental findings. For example, eye-movement training not only induces plasticity within oculomotor brain areas but it also alters brain activation in the striate and extrastriate cortex (Nelles et al., 2007, 2009, 2010), i.e., in areas where VRT was also observed to induce activation changes (Marshall et al., 2008). In line, Kerkhoff et al. (1992) observed a visual field border shift following a period of VST, raising the question of to what extent the functional improvements in compensatory training may, in fact, be at least in part the consequence of vision restoration. As far as AViST is concerned, the retino-colliculo-extrastriate pathway is a possible neural substrate mediating its visual exploration and oculomotor improvements. Indeed, the colliculo-extrastriate pathway is crucial in integrating audio-visual information in humans (for a review: Stein and Stanford, 2008) and is known to be functionally spared in patients with Primary Visual Cortex (V1) lesions (Tamietto et al., 2012). In line, recent evidence in animals suggests that a systematic audio-visual training can reinstate visual behavior in hemianopic cats, after a lesion to the striate cortex (Jiang et al., 2015). Crucially, such recovery co-occurs with the reinstatement of visual responsiveness in deep layer neurons of the ipsilesional SC. Therefore, audio-visual stimulation may enhance activity within this spared network, and recruit additional cortical areas responsible for oculomotor planning, such as the frontal eye fields, which are known to be strongly connected to the SC and to be involved in spatial orienting behaviors (for a review: Krauzlis et al., 2013). However, similar to VST, and in addition to network-level plasticity, AViST might also elicit neural restoration in the occipital cortex, since eye movements are known to lower the perceptual threshold. Indeed, both in primates and humans, the visual system uses saccades as a preferred sampling strategy (Martinez-Conde et al., 2004, 2009; Otero-Millan et al., 2008; Troncoso et al., 2008; Rolfs, 2009), which allows more efficient sampling of fine spatial detail (Donner and Hemilä, 2007) and elicits stronger responses in V1 neurons (Martinez-Conde et al., 2000, 2002; Herrington et al., 2009). In fact, this point can also be made with regard to VST.

In any event, the neural correlates underlying improvements after each form of treatment need further investigation, which would be relevant both from a theoretical and clinical point of view. Theoretically, the investigation of the neural bases of visual field recovery might provide more useful information about neural plasticity mechanisms after lesions. From a within-systems plasticity perspective, given the diversity observed in patients with brain lesions, it is important to know the location and magnitude of intact neuronal tissue required for different treatment modalities to have a positive effect. Similarly, at a network plasticity level, it is imperative to understand what functional network level circuitry is necessary to assist with training effects or other stimulation approaches such as those using alternating currents (Bola et al., 2014). At the clinical level, this knowledge will be useful to predict the outcome of each type of treatment. Based on this understanding one could choose the most effective treatment procedure for individual patients, possibly incorporating a combination of treatments, with the aim of optimizing improvements in visual rehabilitation.

In addition, attentional processes and the mechanisms regulating training improvements in VST, AViST and VRT deserve special interest, as they appear to serve as a key area of theoretical overlap between the treatment approaches. Presumably, HVFD patients typically direct their focus of attention to the intact field, reinforcing an attention pattern that favors the intact field section and ignores ARV. Shifting attention towards the intact field might, on the one hand, reduce neural activation in partially defective regions of the visual cortex, i.e., ARV, while, on the other hand, oculomotor exploration of the blind field may diminish. Indeed, attentional cues presented in the blind field boost the effects of VRT (Poggel et al., 2004), suggesting that attention potentiates visual rehabilitation. In this study, a special cueing procedure was administered during VRT to help patients shift their focus of attention towards a certain area located at the visual field border and deeper in the blind field. Visual detection improved especially in those parts of the visual field where the cue was presented (Poggel et al., 2004).

Similarly, recent EEG evidence (Dundon et al., in press), collected before and after AViST, suggests that an attention shift occurs during the training. In addition to improvements on the previously listed behavioral measures, AViST also drove a reduction in P300 components in response to stimuli presented in the healthy field during a simple visual detection task. This neurophysiological effect likely reflects a reduced allocation of attention towards the intact visual field after training. Interestingly, Marshall et al. (2008) who studied BOLD change following VRT, noticed activation reductions in the right inferior and middle temporal, medial frontal, and bilateral basal ganglia, when a group of right-hemianopic (i.e., intact right hemisphere) participants responded to stimuli in their healthy visual field. No reductions occurred in the left hemisphere, suggesting that activation reductions appeared to be restricted to the healthy hemisphere. Promising early signs of network-level neuroplastic overlap between VRT and AViST are therefore emerging, specifically within the domain of attentional rebalancing.

Another area of interest for future research is the application of non-invasive brain stimulation to boost the efficacy of the rehabilitative techniques. Emerging evidence supports the efficacy of using non-invasive brain current stimulation in the treatment of visual impairments after optic nerve lesions and HVFD (Halko et al., 2011; Sabel et al., 2011a; Plow et al., 2012; Gall et al., 2013; Schmidt et al., 2013). Recent studies have demonstrated that combining VRT with transcranial direct current stimulation (tDCS) applied to posterior occipital regions may enhance the effect of VRT without tDCS (Plow et al., 2012). In the case of tDCS, stimulation appears to give impetus to excitability changes in visual cortex and other brain structures (Antal et al., 2006). Studies using EEG power-spectra analysis have described significant increases in alpha-activity, localized to occipital sites, following repetitive, transorbital alternating current stimulation (rtACS; Schmidt et al., 2013). Non-invasive stimulation, therefore, seems to elicit increased neuronal network synchronization which is substantiated by lasting bilateral synchronous waves in alpha and theta ranges in central and occipital brain areas (Sabel et al., 2011a) and restore lost functional connectivity networks in the brain (Bola et al., 2014). Concerning VRT as an independent method, it still needs to be shown whether neuronal network synchronization, i.e., increases of spectral coherence in the visual cortex but also in wider distributed networks, serves as a mechanism of action.

In summary, the field of visual rehabilitation is at a promising junction. Both compensatory and restorative technologies have become available for the treatment of HVFD and possibly other types of visual field defects. It will be important to further delineate what the common elements between the approaches are, and what makes each one unique. Furthermore, the techniques should be standardized to compare results between laboratories and results should be made available to the medical community to ascertain best practice clinical care.

Importantly, the three reviewed approaches differ in terms of time on task (intensity) and duration. Indeed, VST training procedures usually last 5–6 weeks with daily 1 h sessions, the AViST training has a duration of 2 weeks with daily 4 h sessions, while the VRT approach consists of a 3–6 months training with daily 30 min sessions. Despite these duration differences, they each operate within an adaptive framework. Recent evidence supports adaptive treatments in order to ensure that patients are consistently challenged without being overly frustrated or fatigued by task demands, which is optimal for both maximising clinical outcome and avoiding patient drop outs (Klingberg, 2010). Indeed, the reported drop-out rates of these approaches seem to be negligible: Pambakian et al. (2004) reported a drop-out of two out of 29 patients during a VST training, due to aggravated clinical and social conditions, while the other studies do not mention any case of drop-outs.

Broadly speaking, visual rehabilitation targeting restoration of a portion of the visual field, seems to represent an optimal approach to address visual field function and size. However, VRT consists of a long-lasting training protocol, which may not suit the life circumstances of all patients. Although there were no reports of drop outs or an extremely low rate in those VRT studies that were conducted in a laboratory setting (e.g., Kasten et al., 1998; Mueller et al., 2007; Gudlin et al., 2008), clinical experience dictates that time-consuming training protocols may constitute a reason for dropping out in some patients. Here, faster methods of non-invasive brain stimulation may offer a complementary or alternative solution. In addition, from an ecological perspective, improvements at the visual field border may not be sufficient to completely recover impairments in daily life activities. For those still suffering everyday life impariments despite having been treated with restoration techniques, compensatory approaches, such as VST or AViST, might help overcome these limitations.

Only by considering evidence from all fields of study, and employing an open, critical debate, can we make the fastest possible progress to help patients with partial blindness. Further, we should not simply consider local events at the lesion site or immediately around it, but also study the visual system in a holistic manner, including global brain network function, saccade-induced facilitations, cross- and/or multimodal influences and attentional mechanisms. Thus, by considering the topic in a holistic way we can serve both research needs and clinical necessities in a manner that is not microscopic but macro-scopic with the ultimate goal to optimize clinical care in vision rehabilitation.

## Conflict of Interest Statement

The authors declare that the research was conducted in the absence of any commercial or financial relationships that could be construed as a potential conflict of interest.
